# Evaluation of Physical Risk during Necropsy and Morgue Activities as Risk Management Strategy

**DOI:** 10.3390/ijerph18168266

**Published:** 2021-08-04

**Authors:** Zoe Del Fante, Nicola Di Fazio, Adriano Papale, Paola Tomao, Fabio Del Duca, Paola Frati, Vittorio Fineschi

**Affiliations:** 1 Department of Anatomical, Histological, Forensic and Orthopaedic Science, Sapienza University of Rome, Viale Regina Elena 336, 00161 Rome, Italy; zoe.delfante@uniroma1.it (Z.D.F.); nicola.difazio@uniroma1.it (N.D.F.); fabio.delduca@uniroma1.it (F.D.D.); paola.frati@uniroma1.it (P.F.); 2 Department of Occupational and Environmental Medicine, Epidemiology and Hygiene, Italian Workers’ Compensation Authority (INAIL), Monte Porzio Catone, 00078 Rome, Italy; a.papale@inail.it (A.P.); p.tomao@inail.it (P.T.); 3 Istituto di Ricovero e Cura a Carattere Scientifico (IRCCS) Neuromed, Via Atinense 18, 86077 Pozzilli, Italy

**Keywords:** risk management, physical risk, autopsy

## Abstract

Physical risk assessments allow us to understand work-related critical issues, thus representing a useful tool in risk management strategies. In particular, our study focuses on the identification of already known and emerging physical risks related to necropsy and morgue activities, as well as crime scene investigations. The aim of our study is, therefore, to identify objective elements in order to quantify exposure to such risk factors among healthcare professionals and working personnel. For the research of potentially at-risk activities, data from the Morgue of Policlinico Umberto I Hospital in Rome were used. The scientific literature has been reviewed in order to assess the risks associated with morgue activity. Measurements were performed on previously scheduled days, in collaboration with the activities of different research units. The identified areas of risk were: microclimate; exposure to noise and vibrations; postural and biomechanical aspects of necropsy activities. The obtained results make it possible to detect interindividual variability in exposure to many of the aforementioned risk factors. In particular, the assessment of microclimate did not show significant results. On the contrary, exposure to vibrations and biomechanical aspects of load handling have shown potential risk profiles. For this reason, both profiles have been identified as possible action targets for risk management strategies.

## 1. Introduction

Crime-scene investigation, necropsy and morgue activities are characterized by exposure to different types of risk for the various professional figures involved [1]. Assessment of chemical, physical and biological risk allows us to understand the dangers related to operating procedures [2]. Through specific risk factor assessments [3], which are poorly studied at present, these procedures can be improved, with the adoption of precautions and protections.

From this perspective, clinical risk management identifies potential dangers/threats to which healthcare facilities are subjected, defines and quantifies hypothetical risk scenarios and formulates the most suitable countermeasures, following well-defined strategic corporate objectives.

The main purpose of our study is to identify the risks that are already present [4,5] and those that are emerging in necropsy–morgue activities and on-site inspections.

Moreover, the authors aim to identify objective elements to quantify exposure to physical risk factors related to the workplace. A further step of the study is to provide systematic information on autopsy management that is useful for preventive strategies development.

A quantitative evaluation of major risk factors resulting from exposure to physical agents is performed. Our purpose is to measure exposure to physical risk factors such as noise, vibrations, dust and mechanical stress on the spine and joints. Risk analysis also takes environmental factors into account, studying the microclimate [6] (temperature, humidity, air speed, etc.) and incongruous posture assumed by operators [7]. At present, the literature on risk assessment during necropsy and autoptic investigation is scarce or absent.

In conclusion, the role of the analyzed operators is characterized by an important heterogeneity of tasks, all of which were subject of our study’s monitoring campaign. From the available data, we can conclude that this work scenario is organized in a suitable way in order to minimize operators’ exposure to risks.

## 2. Materials and Methods

Our study involved several complex operating units and research units belonging to Policlinico Umberto I Hospital, each one characterized by a specific role, in order to carry out a correct and complete assessment of different types of risk.

Research was conducted through the examination of the scientific literature on risk assessment possibly associated with morgue activity in order to identify those that had already been investigated [1].

For this purpose, regarding the activity of the Unit of Forensic Medicine, the dissection room and annexed premises of Policlinico Umberto I in Rome were studied.

For the collection and analysis of potentially at-risk activities, cases available during scheduled days at the Morgue of Policlinico Umberto I in Rome were used, including both cadavers available to the Judicial Authority and cadavers destined for diagnostic purposes.

In particular, taking the different risk categories reported into account, homogeneous groups of workers involved in necropsy activities are exposed to:

- Falls in the event of irregularities in external terrain or due to the presence of fixed staircases;
- Loss of balance in particularly cramped closed environments (corridors, flat floors, etc.);
- Falls during night shifts in areas with little or no light;
- Impact or crushing resulting from the possible fall of the transported load;
- Wounds from handling sharp materials (saws, scalpels, surgical forceps, etc.);
- Electric shocks connected to portable equipment powered by electricity;
- Accidents related to the use of equipment and manual tools;
- Exposure to bad weather and/or extreme climatic conditions for all inspection activities carried out outdoors.

After careful review of the scientific literature on the subject, a plan was prepared to monitor what, according to the reviewed authors and department members’ professional experience, were the activities and maneuvers considered to have the most risk of possible consequences for the physical and mental health of workers.

On the basis of these observations, a series of monitoring activities were prepared, partly aimed at assessing individual risk and partly assessing combined risk factors.

Monitoring was conducted by various research units which, through the use of specific methods, participated in scheduled survey sessions during necropsy examinations, with regard to the different examined research types.

Subsequently, we proceeded to identify homogeneous groups of subjects that were at risk including workers in necropsy and morgue services, such as mortuary auxiliary staff, forensic pathologists (teaching and contracted staff), magistrates, resident students, dissection room technicians, radiology technicians, judicial police officers and agents.

Finally, physical risk was identified in those activities carried out within the morgue by the involved personnel, giving priority to health workers directly involved in physical activities:

- Transfer of bodies to the morgue;
- Insertion and removal of bodies in refrigerated storage rooms;
- Medico-legal activities and diagnostic findings (external examinations, autopsies and sampling).

### 2.1. Microclimate

Measurements were made with a microclimatic control unit (HD 32.3) (Delta OHM S.r.l., GHM Group, Caselle di Selvazzano, Italy) equipped with dedicated software and an air and psychrometric temperature probe (HP 3217.2) (Delta OHM S.r.l., GHM Group, Caselle di Selvazzano, Italy), anemometric probe (AP 3203.2) (Delta OHM S.r.l., GHM Group, Caselle di Selvazzano, Italy) and globe-thermometric probe (TP 3276.2) (Delta OHM S.r.l., GHM Group, Caselle di Selvazzano, Italy).

The microclimatic control unit returns data as time function average values, expressed in °C, RH%, m/s, PMV (−3–+3) and PPD (%), respectively.

The assessment of microclimatic conditions was carried out as recommended by legislation and reference guidelines [8,9,10,11] and the collection of objective data through indices of thermal comfort/discomfort calculations [12] (indices of Fanger [11], presumed mean value (PMV) and predicted percentage of dissatisfaction (PPD), which were then compared with what was recommended. The control unit was positioned in a representative point of the monitored environment and was set with clothing thermal insulation (CLO) and level of metabolic activity (MET) values, equal to 1.11 and 1.72, respectively, defined based on observations made during environmental monitoring and indications given by the guidelines.

### 2.2. Noise Exposure

Measurements were made with a SVAN 971 Class 1 integrating sound level meter (Svantek Italia S.r.l., Melzo, Italy) equipped with dedicated software and a SVANTEK SV 18 Preamplifier (Svantek Italia S.r.l., Melzo, Italy) and Delta OHM HD2010UC/A Class 1 integrating sound level meter (Delta OHM S.r.l., GHM Group, Caselle di Selvazzano, Italy) equipped with dedicated software and Delta Ohm HD2010PNE2 Preamplifier (Delta OHM S.r.l., GHM Group, Caselle di Selvazzano, Italy) [12]. Exposure limits have been the subject of several discussions by companies using similar safeguards [13] to assess the risks of developing diseases. In this study, indications from current European directives were followed [14].

### 2.3. Vibration Exposure

Measurements of vibrations transmitted to a hand–arm system were performed with a model SV 103 SVANTEK personal dosimeter and model SV 107SVANTEK triaxial accelerometer. The instrument used was in class 1 and complies with technical specifications of the ISO 8041: 2005, ISO standards 5349-1: 2001 and ISO 5349-2: 2001. Measurements were carried out simultaneously on three orthogonal axes, positioning the accelerometers according to a coordinate system defined by the ISO 5349-1 standard and weighted in frequency through instrumental filters.

The limits established by Italian law on work safety, normalized to a reference period of 8 h, are shown below [15] (Table 1).

The interpretation of results, based on these two reference levels, identifies three classes of risk [16]: below action value; between action value and limit value; and above limit value. These limits are associated with a possible intervention in worker training, environmental reclamation or immediate actions, to reduce exposure levels the same way that risks are reduced in other forms of work [17].

### 2.4. Postural and Biomechanical Aspects of Necropsy Activities

#### 2.4.1. Work Posture Analysis

The assessment of some work phases and operator’s relative postures was carried out in order to understand whether the Rapid Entire Body Assessment (REBA) method [18] is reliable and practicable to evaluate the correlation between postures and musculoskeletal disease risk, as proposed by similar studies [19]. The REBA was chosen because of its effectiveness in assessing both static and dynamic work [20]. This observational method, through special checklists, enables the assignment of a score to the posture of each body part.

#### 2.4.2. Evaluation of Manual Load Handling Activities

The 3D Static Strength Prediction Program software (3DSSPP) [21] developed by the University of Michigan (Ann Arbor, MI, USA), was used for the evaluation of manual load handling activities. It is listed in SIMLII “Guidelines for prevention of disorders and diseases caused by manual handling of loads” among those that are reliable for the assessment of biomechanical risk.

The software calculates the force discharged at the level of L4–L5 lumbosacral junction [22], the population percentage able to perform the specific task without suffering damage for each body district analyzed (wrist, elbow, shoulder, trunk, hip, knee and ankle) [23] and operator stability conditions. Through 10, 20 and 30 kg loads, the handling of the corpse from litter to the dissection table was simulated. Muscle activity was recorded with an 8-channel Wi-Fi surface electromyograph (FreeEMG [24], BTS SpA, Milan, Italy) with a sampling rate of 1 kHz. Electrodes were positioned following the European Recommendations for Surface Electromyography SENIAM [25] and Atlas of Innervation Zones [26]. Erector spinae, superior trapezius, middle trapezius and anterior deltoid muscles electromyographic signals were acquired bilaterally, thus analyzing muscle involvement during each movement.

## 3. Results

### 3.1. Micoclimatic Conditions

**Figure 1:**
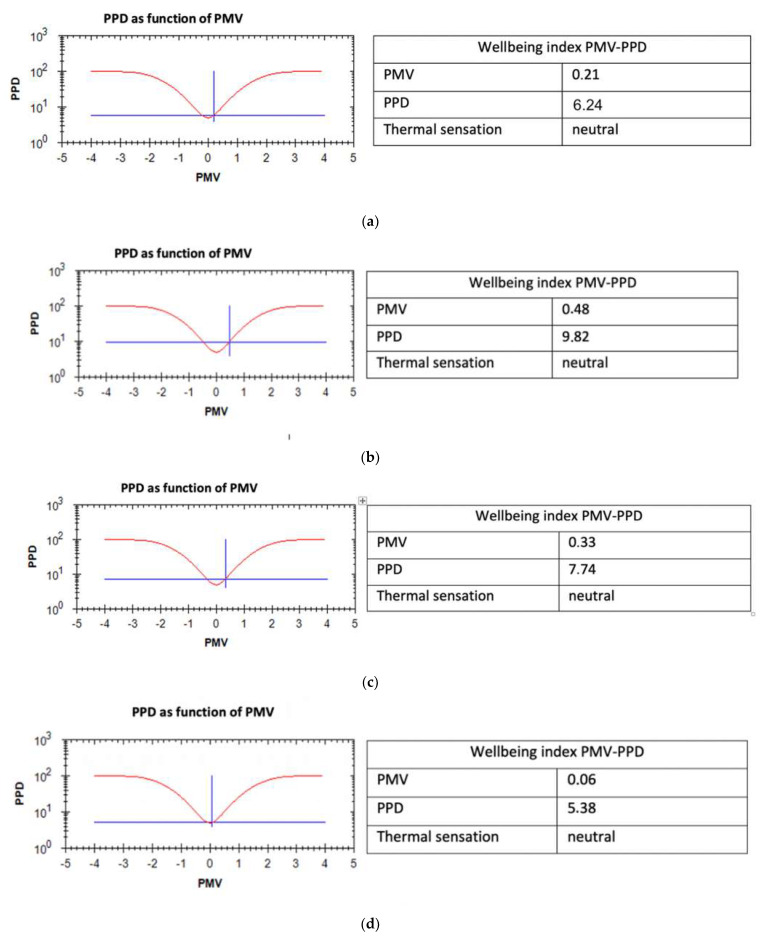

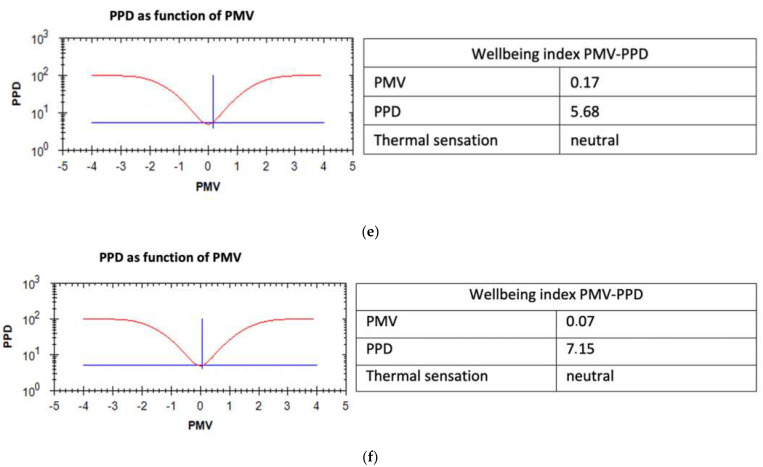
PMV (presumed mean value) and PPD (presumed percentage of dissatisfied) as detected on: (**a**) 24.09.2016; (**b**) 29.09.2016; (**c**) 30.09.2016; (**d**) 06.10.2016; (**e**) 06.12.2016; (**f**) 07.12.2016.

Environmental monitoring always detected temperatures slightly lower than those recommended by the guidelines. Similarly, air speed was slightly lower than recommended values. During the first four monitoring days relative humidity was higher than recommended values. However, microclimate evaluation based on PMV and PPD calculations defined the environment as thermally acceptable during all monitoring days (Figure 1).

The results of measurements carried out during monitoring activities are summarized in the table below (Table 2).

### 3.2. Exposure to Noise

**Figure 2:**
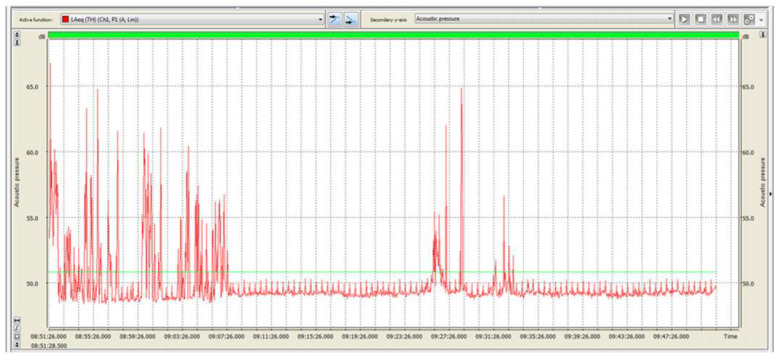
24.09.2016. Acoustic emissions in dissection room, detected in activity-free intervals with functioning air conditioning system. Red line: level during T period of 15” (Leg), expressed as dB (A). Green line: equivalent sound level during the entire monitoring period (Leg), expressed as dB (A).

**Figure 3:**
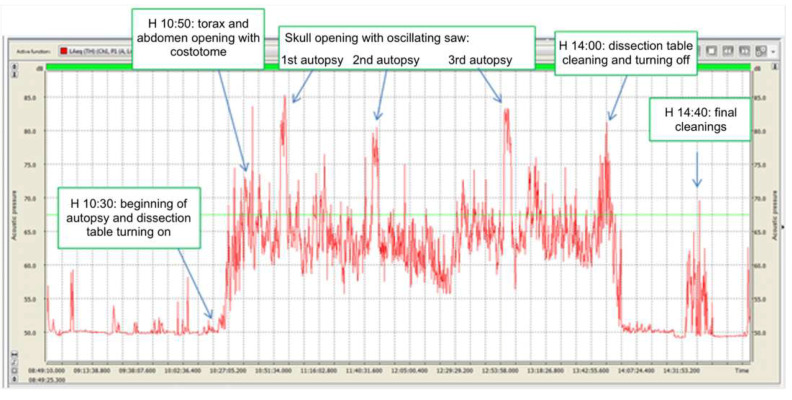
30.09.2016. Acoustic emissions during 3 autopsies, each one performed on a different dissection table. Red line: equivalent sound level during T period of 15” (Leg), expressed as dB (A). Green line: equivalent sound level during the entire monitoring period (Leg), expressed as dB (A).

**Figure 4:**
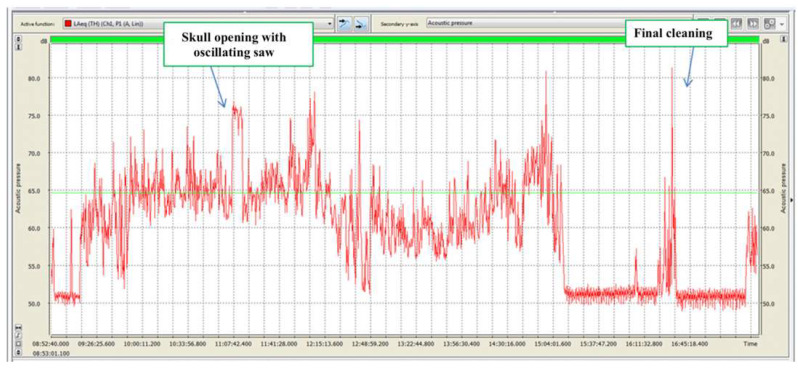
06.10.2016. Acoustic emissions during autopsy. Red line: equivalent sound level during T period of 15” (Leg), expressed as dB (A). Green line: equivalent sound level during the entire monitoring period (Leg), expressed as dB (A).

**Figure 5:**
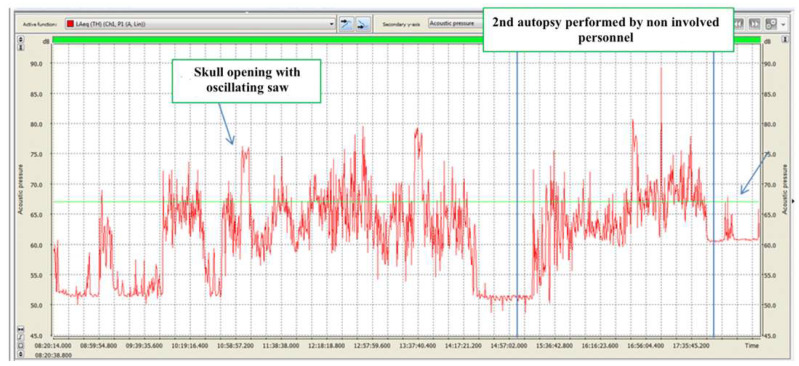
07.10.2016. Acoustic emissions during autopsy. Red line: equivalent sound level during T period of 15” (Leg), expressed as dB (A). Green line: equivalent sound level during the entire monitoring period (Leg), expressed as dB (A).

With regard to noise exposure, it should be specified that each dissection room operator participates in one or more autopsies daily, with each one extremely variable in duration. During autopsies, different noise-generating devices and instruments are generally used with different methods and durations. It was, therefore, impossible to identify homogeneous groups of exposed workers on which to carry out the exposure assessment based on time and specific job. Consequently, the measurements carried out relate to the contribution of dissection room activities to general noise exposure, as listed below (Table 3) (Figure 2, Figure 3, Figure 4 and Figure 5).

The identified noise sources were: dissection table mechanism, oscillating saw, rib-cage scissors, glassware washer, cleaning equipment and air conditioning system. Particular attention was paid to excessive noise produced by the oscillating saw and the study of its risk factors, as evaluated in other areas [27].

Daily exposure level (LEX8h dB (A)), therefore, refers to operators’ exposure considering their theoretical range of 8 h activity in the dissection room. Comparing data with regulatory limits shows equivalent levels are much lower than the inferior action limit. Peak levels were also found to be below the lower action limit.

### 3.3. Exposure to Vibrations

**Figure 6:**
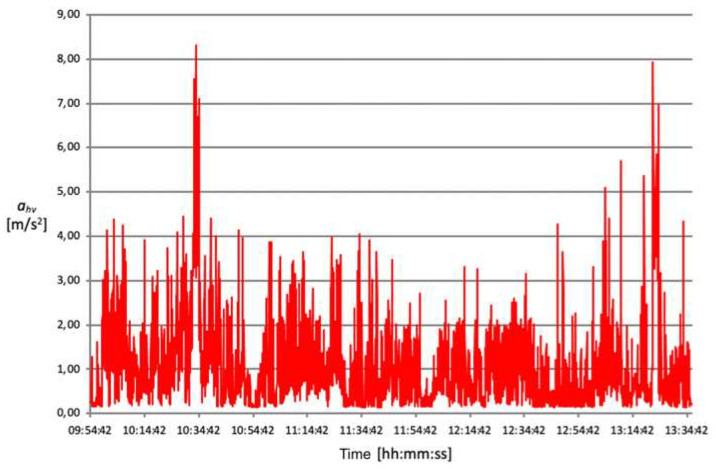
Acceleration root mean square distribution over time, detected on 06.12.2016.

**Figure 7:**
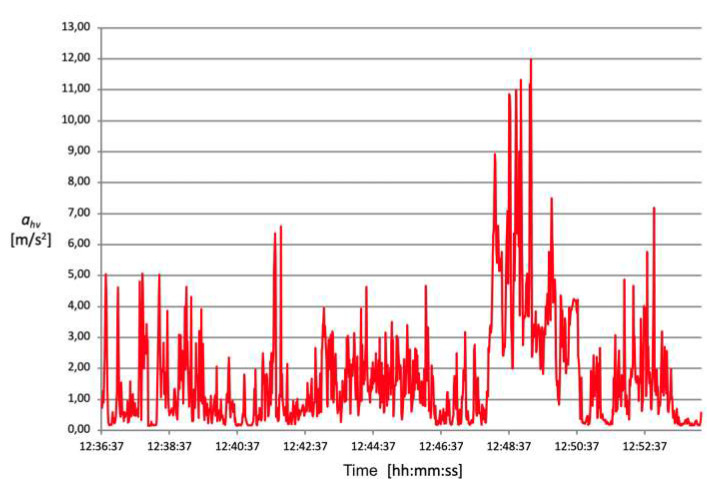
Acceleration root mean square distribution over time, detected on 07.12.2016 registration n. 2.

**Figure 8:**
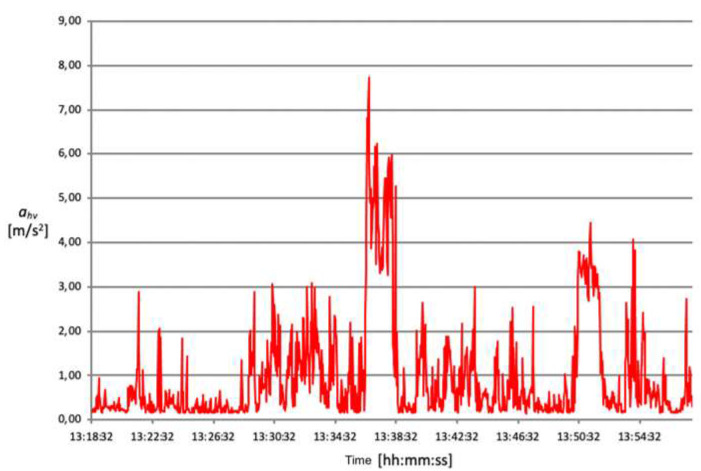
Acceleration root mean square distribution over time, detected on 07.12.2016 registration n. 3.

**Figure 9:**
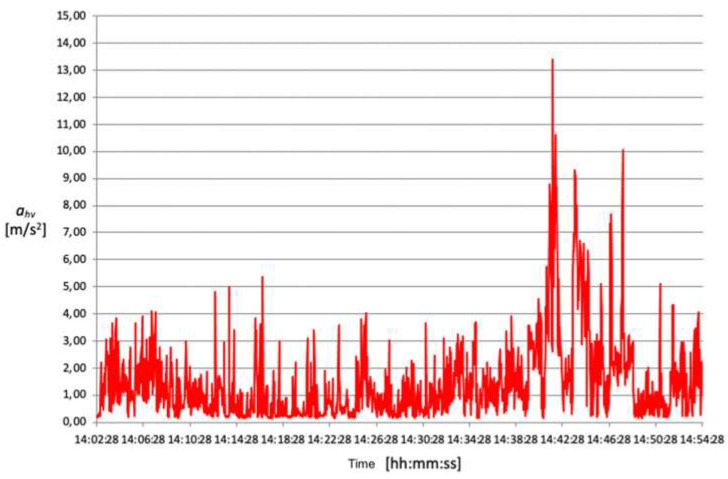
Acceleration root mean square distribution over time, detected on 07.12.2016, registration n. 4.

The acceleration mean square value (ahv) transmitted from the oscillating saw to the operator’s hand–arm system during the opening of the cranium theca is between approximately 4 and 5 m/s^2^ (Figure 6, Figure 7, Figure 8 and Figure 9).

These data demonstrate that, despite intersubjective variables consisting mainly of height, posture and applied force, the detected exposure values are similar. Conversely, the time required to perform a complete cranium cut ranges between 2 and 8 min, therefore varying significantly from one technician to another. This results in different values of vibration risk exposure depending on the duration of the procedure. Exposure time plays a fundamental role in the final calculation and, consequently, on the data’s compliance with action values.

The root mean square value of accelerations (ahv) measured during bone harvesting operation is just under 3 m/s^2^ for an exposure time of approximately 2 min.

Another complex evaluation refers to estimating the average time and vibration value for each activity operators perform without an oscillating saw. Generally, the duration of a complete autopsy is approximately one hour, while the acceleration’s mean square value (ahv) measured lies approximatively between 0.9 and 1.6 m/s^2^.

Table 4 shows the results relating to the exposure level of daily vibrations (A (8)) and relative risk classes, respectively.

### 3.4. Postural and Biomechanical Aspects of Necropsy Activities

The measurements made in order to study the postural and biomechanical aspects of necropsy activities produced suggestive data about the ergonomics of medical sector activities, documenting a general increase in biomechanical load for the upper limbs and spine. 

Postures assumed during scalp incision and overturning, thorax and abdomen incision, cutaneous and muscular planes disconnection and evisceration obtained a score between 8 and 10, according to the Rapid Entire Body Assessment (REBA) method, therefore requiring the adoption of preventive measures. 

Postures assumed during craniotomy, associated with the way the oscillating saw is held and the vibrations transmitted to the hand–arm system, obtained the highest REBA score (score 11), classified in the highest risk class [28]. This risk class is associated with increased likelihood of musculoskeletal disease [29]. 

On the other hand, postures assumed during organ section and coxo-femoral joint exploration (an activity carried out only occasionally), do not require torsional movements of the trunk, thus obtaining a lower REBA score, between 4 and 7, identified as medium–high risk class with a medium-term need for preventive interventions.

The evaluation of manual load handling according to the 3D Static Strength Prediction Program software (3DSSPP) showed that biomechanical overload resulting from transferring the corpse from litter to the dissection table is greater during the final phase of handling (deposit on the dissection table) than during the initial phase. In fact, during the initial phase of handling, the force discharged at L4-L5 levels went from 2856 N handling 10 kg to 4895 N handling 30 kg, while in the final phase, values went from 3063 N handling 10 kg to 5182 N handling 30 kg. With regard to the percentage of the population able to carry out this task without suffering damage in various body districts, the simulation with 30 kg registered values ranging from 19% for the wrist (red risk range), 27% for the shoulders (risk range borderline between yellow and red) and 22% for the trunk (red risk range) during the initial phase, to 18% for the wrist (red risk range), 26% for the shoulders (red risk range) and 17% for the trunk (red risk range) in the final phase of lifting. 

Electromyographic measurements during the transfer of the corpse from litter to the dissection table documented a biomechanical overload of the upper limbs and rachis, as evidenced by significant levels of activation of both deltoid muscles and erectors of the column, particularly on the side towards which the transfer was carried out.

## 4. Discussion

Risk management in healthcare settings seeks to identify tools to assess and govern the risks inherent in these activities, pursue improvements in the overall management system, develop efficient tools and identify moral and economic consequences arising from patients’ or operators’ exposure to risk itself.

By their nature, medical procedures involve risks., Although the allowed error margins are quite narrow, these can be classified as:

- Risks to inpatients/caregivers related to clinical care activities (clinical risk);
- Environmental safety: physical state of the structure, fire, explosions, radiation, noise, magnetic fields (environmental risk);
- Personnel safety, which includes environmental risks and those related to the activity performed: infections, occupational diseases (worker protection);
- Economic and financial risks resulting from the activity carried out (insurance).

All of these aspects must be addressed within a health facility’s risk management program, in a comprehensive and integrated manner, while respecting specific skills and responsibilities.

In the prevention of undesirable events, Risk Management in Healthcare is one of the most effective tools, although it has historically been characterized mainly by a “defensive” approach, aimed at reducing the frequency of professional accidents and damage to patients, possible claims and resulting costs.

A key part of this process is risk identification, i.e., the correct identification of situations, behaviors and procedures which can lead to a “loss”.

Sources of risk identification are numerous; some of the most relevant include:

- Safety of environments and equipment;
- Infection control pathways;
- Quality improvement programs and accreditation procedures;
- Surveillance of the degree of satisfaction and employees’ and users’ complaints;
- Adverse events: professional negligence, accidents, injuries to users and visitors, compensation for damages, etc.

The approach is based on the assumption that every error represents the consequence of the preceding problems, which may become apparent even before the adverse event occurs.

As a consequence, in order to reduce errors, rather than acting restrictively on dependents, it is preferable to look for solutions in the system.

The aim of our study is, therefore, to identify the environmental risks of the dissection room, with particular reference to the assessment of the physical risks to which operators are exposed.

The various Research Units involved have characterized and monitored all possible risks related to autopsy activities. Attention was focused on specific manual skills and operational maneuvers performed during the activity and on the peculiarities of those activities carried out during judicial inspection.

Monitoring campaigns conducted between September and December 2016 demonstrated the substantial adequacy of the assessed environment with respect to the recommended values for all parameters under investigation. Measurements performed for the assessment of microclimate documented the adequacy of parameters with respect to internationally recognized reference values [30]. 

Evaluations of vibration exposure documented important differences in exposure dose based on gender and operator experience; in fact, female and younger operators appear to be more exposed to vibration, probably due to their having less training. The data show significant exposure to vibration that must be contained through remediation of the preside, in order to avoid the development of syndromes such as hand–arm syndrome [31].

Analysis of the postural and biomechanical aspects of necropsy activities through observation of activity characteristics, environment and work tools used allowed us to define the type of activity carried out, detailing its phases, related postural and biomechanical aspects and related risks for the operator’s musculoskeletal system. Autopsy investigation execution implies a considerable variability in activities and their duration, as well as in the postures assumed by the operators in order to adapt to the activity carried out, in line with the previous literature [32]. The necessity to adapt posture to specific autoptic phases often implies the choice of incongruent postures and movements that produce an increase in biomechanical overload of the upper limbs and spine.

## 5. Conclusions

The results of the investigation conducted at Policlinico Umberto I of Rome showed substantial adequacy of the analyzed parameters compared to what has been established by reference standards. In particular, physical risk associated with necropsy activity was studied in relation to microclimate, noise exposure, vibration exposure and, finally, the postural and biomechanical aspects of these activities. Data analysis revealed an inter-individual exposure variability to many of these risk factors. In particular, exposure to vibration and biomechanical aspects of load-handling present potential risk profiles, thus identifying possible targets for risk management strategies. Similar analyses have been conducted on microbiological and chemical risk assessments associated with morgue activity [33,34,35]. Further developments could lead to the analysis of the workplace safety climate, perceived risk, and the involvement in safety management. The latter plays a pivotal role in risk management since workers’ safety perception determines improvement in work quality and reduces risks [36].

## Figures and Tables

**Table 1:** Limits established by law 81/08 after 2002/44/EC Directive.

	Hand–Arm Vibrations (HAV) A(8)	Full Body Vibrations (WBV) A(8)
Limit value	5 m/s^2^	1.0 m/s^2^
Action values	2.5 m/s^2^	0.5 m/s^2^
Limited period	20 m/s^2^	1.5 m/s^2^

**Table 2:** Microclimatic values: PMV = presumed mean value; PPD = presumed percentage of dissatisfied.

Parameters	Mean ± DS (Min–Max)						Reference Limit *
	24.09.2016	28.09.2016	30.09.2016	06.10.2016	06.12.2016	07.12.2016	
Air temperature (°C)	18.00 ± 0.68 (16.8–18.8)	19.22 ± 0.29 (18.6–19.8)	18.17 ± 0.99 (16.4–19.3)	17.01 ± 0.29 (16.2–17.6)	16.83 ± 0.74 (14.4–18.2)	16.96 ± 2.20 (12.7–19.5)	20–24
Relative humidity (%)	67.51 ± 5.00 (56.1–75.0)	68.14 ± 1.33 (63.6–70.8)	69.04 ± 2.87 (63.8–74.9)	75.75 ± 2.09 (71.3–81.2)	52.43 ± 4.37 (46.2–60.8)	50.04 ± 6.82 (41.2–63.6)	40–60
Air velocity (m/s)	0.16 ± 0.06 (0.01–0.35)	0.02 ± 0.01 (0.00–0.10)	0.02 ± 0.02 (0.00–0.12)	0.02 ± 0.02 (0.00–0.22)	0.04 ± 0.04 (0.00–0.25)	0.03 ± 0.03 (0.00–0.19)	>0.05 **<0.10 ***
PMV	0.21 ± 0.12 (−0.07–0.43)	0.48 ± 0.05 (0.38–0.56)	0.33 ± 0.15 (0.06–0.51)	0.17 ± 0.05 (0.00–0.27)	0.06 ± 0.12 (−0.36–0.31)	0.07 ± 0.32 (−0.58–0.43)	±0.5
PPD (%)	6.24 ± 1.06 (5.00–8.94)	9.82 ± 0.96 (8.05–11.48)	7.74 ± 1.80 (5.07–10.43)	5.68 ± 0.31 (5.00–6.49)	5.38 ± 0.49 (5.00–7.67)	7.15 ± 1.72 (5.00–12.02)	≤10%

* Istituto Superiore per la Prevenzione e la Sicurezza del Lavoro (ISPESL). Linee guida sugli standard di sicurezza e di igiene del lavoro nel reparto operatorio. ISPESL, 2009 [8]. ** Air velocity should not damage exposed working personnel; it should be maintained at lowest possible values (not below 0.05 m/s). *** Istituto Superiore per la Prevenzione e la Sicurezza del Lavoro (ISPESL). Microclima, aerazione e illuminazione nei luoghi di lavoro. ISPESL, 2006 [9].

**Table 3:** Exposure levels measurements to noise sources.

Parameters	24.09.2016	30.09.2016	06.10.2016	07.10.2016	07.12.2016
LEX_8h_ dB(A)	50.8	66.5	65.0	68.1	71.7
L_peak_ dB(C)	103.0	101.1	98.8	108.4	111.7

**Table 4:** Daily vibration exposure levels assessment A (8).

Measure Description	HAV	WBV			Time of Exposure (h/min.)
	a_hv_ (m/s^2^)	1.4 × a_wx_ (m/s^2^)	1.4 × a_wy_ (m/s^2^)	1.0 × a_wz_ (m/s^2^)	
Operations performed without oscillating saw during autopsy	1.622	-	-	-	4 h
Opening of the cranial cavity by oscillating saw	4.960	-	-	-	20 min
Bone harvesting by oscillating saw	2.848	-	-	-	4 min

## Data Availability

Data supporting reported results are available on request.
